# Modulating CD8⁺ T cell immunity through exercise: mechanistic insights and implications for precision immunotherapy

**DOI:** 10.7150/thno.126053

**Published:** 2026-01-01

**Authors:** Xinyuan Zhao, Xu Chen, Pei Lin, Yunfan Lin, Weiyao Feng, Meiyan Zou, Nina Li, Li Cui

**Affiliations:** Stomatological Hospital, School of Stomatology, Southern Medical University, Guangzhou, 510280, Guangdong, China.

**Keywords:** CD8⁺ T cells, physical exercise, immunomodulation, T cell dynamics, immune metabolism

## Abstract

CD8⁺ T lymphocytes are pivotal effectors of adaptive immunity, executing cytotoxic mechanisms essential for pathogen clearance, tumor surveillance and tissue protection. Their activity is shaped by antigenic stimulation, cytokine networks and the metabolic and structural architecture of the tissue microenvironment. Physical exercise has emerged as a potent, non-pharmacological modulator of CD8⁺ T cell biology, capable of influencing recruitment, activation, differentiation and functional persistence. Acute exercise mobilizes effector and memory subsets, enhances trafficking to peripheral tissues and transiently alters activation thresholds, while sustained training remodels subset composition, preserves mitochondrial competence and attenuates immunosenescence. These adaptations are orchestrated through integrated neuroendocrine, vascular and metabolic pathways that recalibrate chemokine gradients, nutrient availability and energetic support. However, the magnitude and direction of these effects are highly context-dependent, varying with host physiology, disease state and microenvironmental constraints. This Review integrates mechanistic and translational evidence across physiological and pathological settings—including cancer, infectious, neurological and metabolic diseases—to clarify when and how exercise can be leveraged to reinforce cytotoxic immunity. We highlight key methodological and biological challenges, and propose biomarker-guided, microenvironment-informed and adaptively titrated exercise interventions as a framework for advancing exercise from an adjunctive measure to a modulatory, precision immunotherapy.

## 1. Introduction

CD8⁺ T lymphocytes are pivotal effectors of adaptive immunity, orchestrating cytotoxic mechanisms essential for the elimination of virally infected cells and the eradication of malignant cells arising through oncogenic transformation 1-3. Upon recognition of antigenic peptides presented by MHC (Major histocompatibility complex) class I molecules, naïve CD8⁺ T cells undergo clonal expansion and acquire effector functions, including the secretion of perforin and granzymes to induce target-cell apoptosis, and the production of pro-inflammatory cytokines such as IFN-γ and TNF-α to amplify immune responses 4, 5. Their functional integrity is sustained through a tightly regulated continuum of activation, proliferation, differentiation and memory formation, governed by co-stimulatory signals from antigen-presenting cells, cytokine-mediated survival and differentiation cues, chemokine-directed trafficking, and metabolic inputs from the tissue microenvironment 6, 7. These processes generate heterogeneous subsets—including short-lived effector cells, central and effector memory populations, and tissue-resident memory T cells—that collectively maintain immune surveillance and mediate rapid protective responses 8, 9. In both physiological and pathological contexts, the magnitude and quality of CD8⁺ T cell responses critically influence the outcome of infection control, tumor surveillance and chronic inflammatory processes 10, 11.

Physical exercise is increasingly recognized as a potent modulator of immune homeostasis, exerting effects that span systemic physiology and local tissue niches to influence multiple aspects of T cell biology 12, 13. Acute bouts of exercise mobilize T cells from lymphoid reservoirs such as the spleen, bone marrow and peripheral lymph nodes into the circulation, primarily through catecholamine-driven β₂-adrenergic receptor signaling that promotes detachment from stromal niches and enhances trafficking to peripheral tissues 14, 15. This mobilization preferentially enriches antigen-experienced effector and effector memory CD8⁺ T cells, transiently boosting cytotoxic surveillance 16, 17. In contrast, chronic exercise training drives longer-term remodeling of the T cell compartment, expanding the naïve pool, increasing central and effector memory subsets with high proliferative potential, and reducing terminally differentiated or senescent-like populations 18. These adaptations are associated with improved proliferative capacity, reduced expression of exhaustion markers such as PD-1, and enhanced cytokine production, reflecting an attenuation of immunosenescence 19.

Over the past decade, both preclinical and clinical studies have expanded our understanding of how exercise shapes CD8⁺ T cell dynamics in health and disease. In healthy individuals, regular moderate-intensity exercise has been linked to increased naïve and central memory CD8⁺ T cell frequencies, improved proliferative capacity, and reduced accumulation of senescent or exhausted phenotypes 20. In oncology, exercise has shown the capacity to remodel the tumor microenvironment (TME), increasing vascular perfusion and, in some models, enhancing tumor infiltration by cytotoxic T cells, thereby augmenting the efficacy of immune checkpoint inhibitors 21, 22. Conversely, other studies have reported negligible or even adverse effects, particularly in immunologically “cold” tumors or in conditions of excessive training stress 23, 24. Such variability underscores the complexity of exercise-immune interactions and suggests that outcomes are contingent upon a multidimensional interplay of exercise parameters, host factors, and the underlying immune-metabolic context.

In this review, we present the first comprehensive synthesis of evidence linking physical exercise to CD8⁺ T cell biology, spanning mechanisms from systemic physiology to tissue-specific immunity. We first analyze how exercise shapes CD8⁺ T cell mobilization, metabolic fitness, and immunosenescence, integrating insights from human studies and preclinical models. The discussion then moves to pathological contexts, examining how exercise shapes CD8⁺ T cell responses in cancer, infectious diseases, neurological disorders and metabolic syndromes, and how the unique immune-metabolic features of each disease microenvironment govern both the magnitude and the direction of these effects. Finally, we critically examine the methodological, mechanistic and translational challenges that currently limit the field, and outline strategies to harness exercise as a precision immunomodulatory intervention optimizing CD8⁺ T cell-mediated immunity in both health and disease.

## 2. Determinants and therapeutic relevance of CD8⁺ T cell function in the immune microenvironment

### 2.1 T cell subsets and their functions within the immune microenvironment

T lymphocytes are a central component of adaptive immunity and can be broadly classified into CD4⁺ and CD8⁺ αβ T cells, γδ T cells, and specialized populations such as regulatory T cells (Tregs), each with distinct developmental mechanisms and effector functions 25, 26. CD4⁺ T cells primarily act as orchestrators of immune responses by providing cytokine-mediated help to other immune cells, supporting antibody production, enhancing antigen-presenting cell function, and optimizing cytotoxic T lymphocyte and natural killer cell activity 27. Through differentiation into subsets such as Th1, Th2, Th17, and T follicular helper cells, CD4⁺ T cells adapt their effector profiles to the nature of the antigenic challenge and the cytokine environment 28. CD8⁺ T cells serve as the primary cytotoxic arm of the adaptive immune system, recognizing antigenic peptides presented on MHC class I molecules and inducing target-cell death through perforin- and granzyme-dependent cytolysis or death receptor signaling, while also releasing pro-inflammatory cytokines that amplify local immune activation 29. γδ T cells occupy an interface between innate and adaptive immunity, capable of rapid responses to stress-induced ligands and metabolites, and contributing to tissue integrity and early immune surveillance 30, 31. Tregs, characterized by the transcription factor FOXP3 (Forkhead box P3) and high CD25 expression, are critical for maintaining peripheral tolerance, controlling immune activation thresholds, and preventing immune-mediated pathology 32, 33.

Within the immune microenvironment, the composition, spatial organization, and activation state of these T cell subsets are dynamically regulated by antigenic stimuli, stromal interactions, chemokine gradients, cytokine networks, and metabolic availability 34. The balance among effector, helper, cytotoxic, and regulatory functions determines the overall immune set point, influencing whether the local environment supports protective immunity, tolerance, or chronic inflammation. Disruption of this balance can occur through altered recruitment patterns, changes in differentiation trajectories, competitive utilization of survival niches, or shifts in the metabolic landscape, ultimately reshaping the hierarchy of T cell subset dominance and modifying the immune response capacity 35, 36.

### 2.2 Determinants of CD8⁺ T cell trafficking, persistence and functional competence

The capacity of CD8⁺ T cells to provide effective immune surveillance is contingent upon a coordinated sequence of trafficking, infiltration, and sustained functional competence within target tissues 2. Entry into affected sites is guided by precisely orchestrated chemokine-chemokine receptor interactions, such as those involving CXCR3- or CCR5-bearing T cells responding to gradients of inflammatory chemokines, and is facilitated by integrin-mediated adhesion to endothelial ligands including ICAM-1 (Intercellular adhesion molecule 1) and VCAM-1 (Vascular cell adhesion molecule 1). The efficiency of this process depends on the alignment of chemotactic cues with permissive vascular and stromal architecture, enabling transendothelial migration and tissue parenchyma access 37. Infiltration can be compromised by structural alterations in vasculature, remodeling of extracellular matrix components, or the establishment of stromal configurations that physically limit lymphocyte entry and dispersal 38.

Once within the target tissue, CD8⁺ T cells must sustain effector activity in a microenvironment that can impose profound metabolic, structural, and immunological constraints. Local hypoxia and restricted nutrient availability limit glycolytic flux and mitochondrial oxidative phosphorylation, impairing the bioenergetic capacity required for proliferation and cytotoxic function 39. Accumulation of metabolic by-products, such as lactate or adenosine, disrupts intracellular signaling pathways, dampens cytokine production, and destabilizes immunological synapse formation 40. Persistent antigenic stimulation can induce a progressive decline in effector potential, manifested as a transcriptionally and epigenetically imprinted state of dysfunction, often accompanied by sustained expression of multiple inhibitory receptors 41. Additional regulatory influences from soluble mediators and cellular constituents of the microenvironment modulate activation thresholds, survival signals, and differentiation trajectories, further shaping the balance between cytotoxicity and tolerance. The convergence of these structural, metabolic, and regulatory factors determines the degree to which CD8⁺ T cells can maintain their surveillance and clearance functions within challenging tissue environments 42.

### 2.3 Immunological and clinical relevance of enhancing CD8⁺ T cell infiltration and function

Restoring or augmenting CD8⁺ T cell function represents a pivotal objective in both fundamental immunology and translational medicine. The ability of these cells to recognize and eliminate aberrant or compromised targets directly influences the balance between immune protection and immune escape, thereby shaping long-term health outcomes. Enhancing their functional capacity encompasses not only increasing cytotoxic potential but also optimizing activation thresholds, proliferative durability, and resistance to dysfunction or exhaustion. At the immunological level, improved CD8⁺ T cell performance can recalibrate effector-to-regulatory cell ratios, reinforce immune surveillance, and maintain the structural and functional integrity of diverse tissues 43. From a clinical perspective, strategies that fine-tune CD8⁺ T cell responses offer the prospect of sustained immune competence, greater adaptability to emerging antigens, and the capacity to modulate inflammation without compromising tolerance. Such approaches must be precisely calibrated to the immune-metabolic context, ensuring that cytotoxic activity is maximized in protective scenarios while avoiding collateral damage in settings where immune restraint is essential. The integration of mechanistic understanding with personalized intervention frameworks holds the potential to translate CD8⁺ T cell optimization into durable improvements in health span and disease resilience.

### 2.4 Linking CD8⁺ T cell biology to exercise immunomodulation

**Figure 1:**
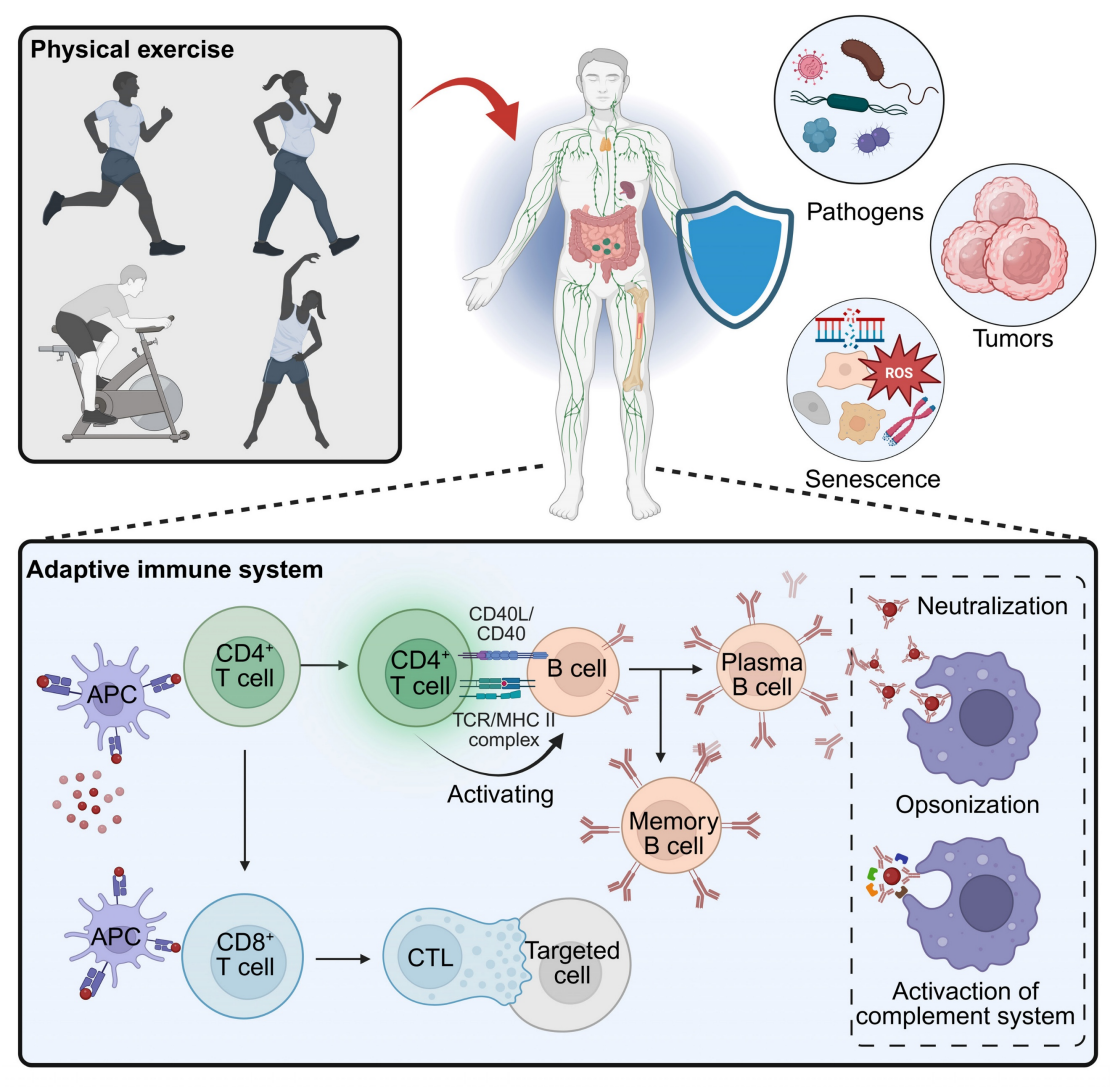
** Exercise remodels adaptive immunity to enhance host defense.** Physical activity elicits systemic cues that strengthen surveillance against pathogens, tumors, and senescent cells. Within the adaptive compartment, exercise optimizes APC-T-cell priming, amplifies CD4⁺ T-cell help for B-cell responses (neutralization, opsonization, complement), and improves CD8⁺ T cell trafficking, persistence, and cytolytic function in target tissues. Created in https://BioRender.com.

Given the pivotal role of CD8⁺ T cells in immune surveillance, pathogen eradication, and the orchestration of tissue-specific immune responses, strategies that enhance their trafficking, metabolic competence, and effector potential hold considerable translational promise 44. Physical exercise emerges as a uniquely multifaceted, non-pharmacological intervention capable of simultaneously modulating systemic physiology and remodeling the immune microenvironment, thereby influencing multiple stages of the CD8⁺ T cell response continuum. Through integrated neuroendocrine activation, vascular remodeling, cytokine redistribution, and modulation of nutrient and oxygen availability, exercise can recalibrate chemokine gradients, adjust activation thresholds, and augment resistance to exhaustion-induced functional decline 45, 46. These adaptations may, in turn, shape clonal expansion dynamics, memory formation, and cytotoxic output, with outcomes highly contingent upon the pre-existing immune-metabolic state of the host and the structural composition of the target tissue microenvironment. Dissecting how exercise-driven systemic signals converge with the molecular pathways underlying CD8⁺ T cell dysfunction—spanning altered antigen presentation, inhibitory receptor signaling, and metabolic stress—across distinct pathological settings will be critical for advancing precision exercise immunotherapy. Such an approach will require integrating high-resolution immune profiling with physiological monitoring to enable biomarker-guided prescription, optimizing cytotoxic T cell immunity while accounting for inter-individual heterogeneity in responsiveness (**Figure 1**).

## 3. Exercise-induced modulating of CD8⁺ T cell immunity under physiological conditions

Exercise functions as a dynamic physiological regulator that modulates CD8⁺ T cell immunity at multiple levels—from transient mobilization and activation to long-term metabolic adaptation and delayed senescence 47, 48. Under physiological conditions, repeated physical activity progressively shapes the functional and metabolic states of cytotoxic T cells, maintaining immune stability while synchronizing immune responsiveness with systemic metabolic demands.

### 3.1 Acute modulating of CD8⁺ T cell mobilization and activation

Acute aerobic exercise modulates rapid redeployment and transient activation of CD8⁺ T cells, allowing the immune system to temporarily enhance cytotoxic surveillance. Transitional memory (TM) CD8⁺ T cells rise immediately and one hour post-exercise, though the increase is smaller than that of effector memory (EM) and EMRA subsets. The preferential mobilization of CD57⁺ CD8⁺ T cells—particularly within EM and EMRA compartments—identifies CD57 as a hallmark of exercise-responsive cytotoxic populations. Likewise, maximal exercise mobilizes peripheral CD8⁺ T cells, especially central memory (KLRG1⁺/CD57⁻) and senescent (KLRG1⁺/CD57⁺) subsets, increasing both frequency and absolute concentration in circulation 49. Beyond redistribution, exercise transiently remodulates functional potential of circulating CD8⁺ T cells. Supramaximal high-intensity interval exercise induces a robust rise in CD8⁺ T cell numbers and strengthens their cytotoxic profile. Despite reduced glycolytic capacity post-exercise, these cells maintain viability and expansion potential, exhibit elevated IFN-γ release, and show enhanced nonspecific cytotoxic activity—evidence of short-term enhancement in immune surveillance 50. A single bout of moderate-to-vigorous aerobic exercise also increases activated CD69⁺ CD8⁺ T cells in young adults, indicating an acute yet self-limited activation phase. While nutrient-sensing pathways in PBMCs remain stable, mitochondrial oxidative capacity increases at the tissue level, reflecting elevated systemic bioenergetic demand 51. Collectively, these observations demonstrate that acute exercise modulates metabolically competent CD8⁺ T cells for transient activation, strengthening immune vigilance without eliciting chronic inflammatory signaling.

### 3.2 Sustained exercise remodulates metabolic fitness of CD8⁺ T cells

Beyond immediate mobilization, habitual exercise modulates long-term metabolic adaptation within the CD8⁺ T cell compartment, shaping their energetic reliance and functional resilience. Regular physical activity enhances mitochondrial dependence while reducing glucose utilization, particularly in effector memory subsets at rest. Upon activation, CD8⁺ T cells from physically active individuals demonstrate greater metabolic flexibility and reduced inflammatory output, such as lower IL-6 expression, compared with less active counterparts 52. These features reflect a sustained remodulating of cytotoxic T cell metabolism that favors oxidative efficiency and limits inflammatory exhaustion. Aerobic fitness correlates positively with mitochondrial mass in naïve CD8⁺ T cells, reflecting enhanced metabolic capacity and energy efficiency 18. Individuals with higher cardiorespiratory fitness display greater mitochondrial respiration and a more pronounced shift toward oxidative phosphorylation, suggesting that exercise-induced metabolic modulating maintains mitochondrial integrity and supports prolonged functional potential 53. Collectively, these data indicate that repeated physiological challenges through exercise act as a metabolic conditioning signal, reinforcing mitochondrial health, sustaining effector competence, and preserving the balanced activation potential of the CD8⁺ T cell pool.

### 3.3 Exercise-mediated delay of CD8⁺ T cell immunosenescence

**Figure 2:**
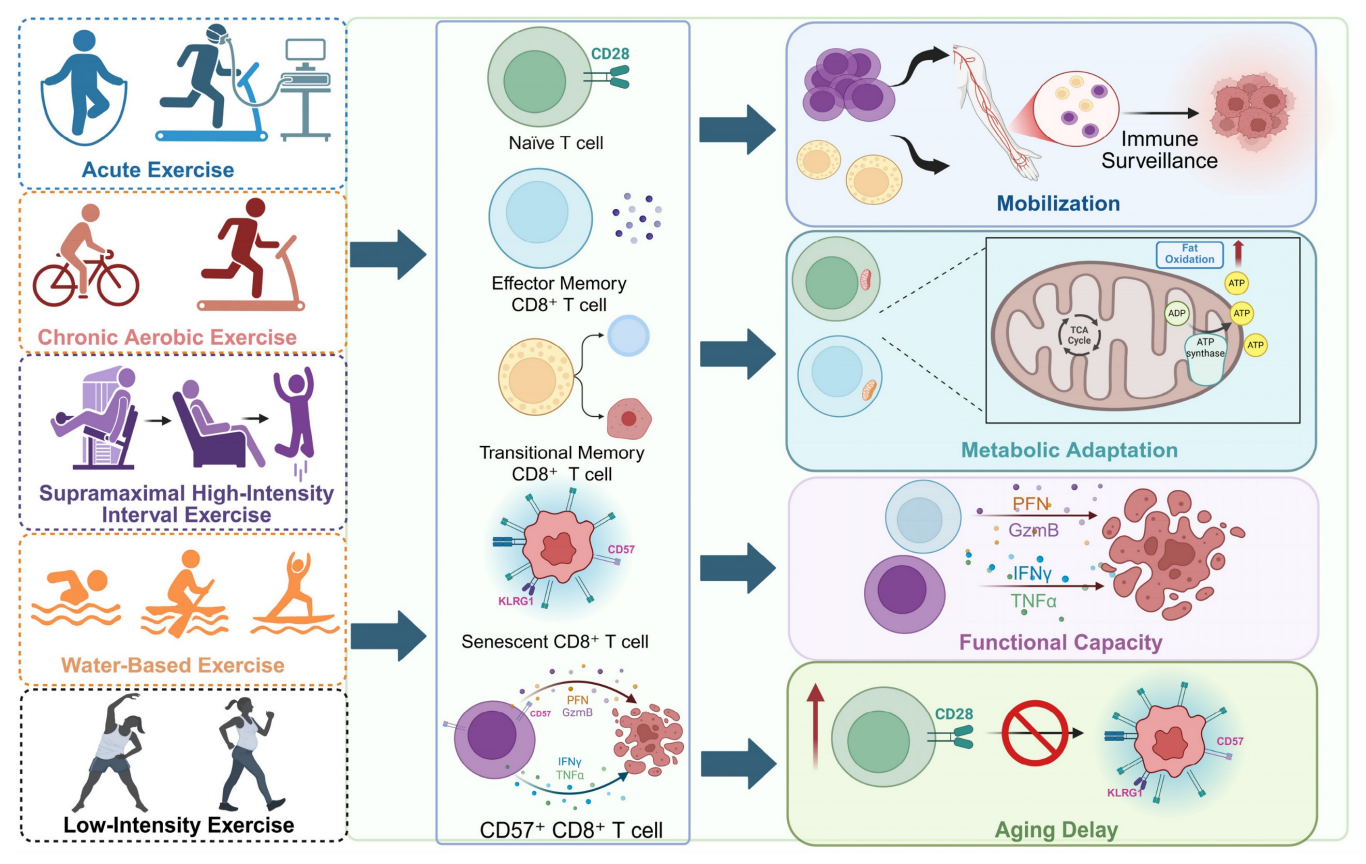
** Exercise shapes CD8⁺ T cell mobilization, metabolic fitness, and immunosenescence.** Different exercise modalities, including acute, chronic aerobic, supramaximal high-intensity interval, water-based, and low-intensity training, modulate CD8⁺ T cell dynamics. Exercise mobilizes naïve, effector, memory, and senescent CD8⁺ T cell subsets, enhances mitochondrial adaptation and cytotoxic mediator release (perforin, granzyme B, IFN-γ, TNF-α), and inhibits the transition of naïve to senescent cells. These effects collectively improve immune surveillance, sustain functional capacity, and delay aging-related decline in T cell immunity. Created in https://BioRender.com.

In addition to optimizing metabolism, exercise actively modulates the aging trajectory of CD8⁺ T cells, shaping their longevity, differentiation balance, and regenerative potential. Sustained physical activity preserves immune competence in older adults by maintaining a balanced CD4⁺/CD8⁺ ratio and supporting a higher proportion of naïve and central memory T cells 54. This remodeling of subset composition reflects a modulatory preservation of immune diversity rather than a passive delay of decline. Active individuals display fewer terminally differentiated effector CD8⁺ T cells, even in CMV-seropositive populations, suggesting that exercise mitigates the cumulative effects of chronic antigenic load on the cytotoxic T cell compartment. Enhanced aerobic performance metrics such as VO₂max and 6-minute walk distance correlate with increased CD4⁺CD45RA⁺ and naïve CD8⁺ T cells, reinforcing the link between systemic fitness and adaptive immune preservation 55. Moreover, moderate aerobic fitness is associated with the maintenance of telomere length in CD28⁺ CD8⁺ T cells, indicating that exercise modulates molecular longevity within the cytotoxic compartment. Individuals with moderate VO₂max exhibit a higher proportion of less differentiated CD8⁺ T cells and longer telomeres, whereas low fitness correlates with accumulation of terminally differentiated effector populations 56. These findings together suggest that physiological exercise recalibrates CD8⁺ T cell differentiation and proliferative capacity through sustained epigenetic and metabolic conditioning, thereby delaying senescence-associated remodeling. Animal studies further substantiate these rejuvenating modulating effects. In aged rats, water-based endurance, resistance, and combined exercise regimens all reduce the proportion of aged and memory CD8⁺ T cells, with combined training most effectively restoring thymic structure and limiting memory CD4⁺ T cell accumulation 57. In humans, low-dose combined resistance and endurance training for six weeks in elderly individuals increases the CD4⁺/CD8⁺ ratio and decreases systemic inflammatory mediators including IL-6, IL-8, IL-10, and VEGF, demonstrating that even modest physical training reestablishes immune homeostasis through functional remodulating of T cell balance 58. Similarly, functional training modalities such as plank or Pilates exercise induce significant remodeling of immune cell composition. Plank exercise enhances CD8⁺ T cell abundance by approximately 28 % after 12 weeks, accompanied by improved respiratory capacity and overall physical performance. Aerobic and low-impact Pilates modulates further decrease immunosuppressive regulatory T cells—including IL-10-producing Tr1 and CD4⁺CD127⁻/loCD25⁺ Tregs—while increasing naïve cytotoxic CD8⁺ T cells 59, 60 (**Figure 2**) (**Table 1**). Collectively, these adaptations demonstrate that consistent exercise imposes a long-term modulating effect on CD8⁺ T cell immunity, rejuvenating the cytotoxic compartment, maintaining proliferative potential, and sustaining immune balance throughout physiological aging.

### 3.4 Integrative mechanisms underlying exercise-induced modulating of CD8⁺ T cell immunity

The capacity of exercise to remodulate CD8⁺ T cell immunity likely arises from the coordinated influence of metabolic, signaling, and epigenetic mechanisms that act across different temporal scales. At the metabolic level, repeated bouts of exercise may enhance mitochondrial biogenesis and oxidative phosphorylation while limiting excessive glycolytic flux 61, 62. Activation of the AMPK-SIRT1-PGC-1α axis might promote mitochondrial turnover, preserve redox balance, and sustain bioenergetic efficiency, thereby preventing the metabolic exhaustion that often accompanies chronic stimulation 63. Exercise-induced fluctuations in NAD⁺ availability and autophagic activity could maintain organelle integrity and ensure adaptive energy supply, establishing a metabolic environment that favors effector persistence and flexible recall responses. At the signaling level, transient endocrine and cytokine oscillations might recalibrate activation thresholds within the CD8⁺ T cell compartment. Periodic surges of catecholamines, IL-7, and IL-15 may transiently engage the PI3K-AKT-mTOR and STAT5 pathways, supporting controlled proliferation and survival while avoiding sustained activation 64, 65.

Such rhythmic modulation appears to reinforce transcriptional modulates associated with stress adaptation and immune balance, potentially acting as a physiological checkpoint that prevents continuous TCR and mTOR engagement, both of which are linked to terminal differentiation and functional decline. At the chromatin level, recurrent metabolic and signaling perturbations might progressively reshape the epigenetic landscape of CD8⁺ T cells. Exercise-driven histone acetylation and DNA methylation changes—mediated in part by NAD⁺-dependent deacetylases such as SIRT1—may stabilize gene-regulatory networks that favor mitochondrial maintenance, DNA repair, and stem-like memory phenotypes. These epigenetic imprints could translate transient metabolic information into durable transcriptional states, preserving effector competence while minimizing inflammatory burden 66, 67. Collectively, these mechanisms suggest that exercise acts as a physiological modulating signal that aligns CD8⁺ T cell immunity with systemic energy metabolism. Through integrated modulation of metabolic flux, signaling thresholds, and chromatin accessibility, regular physical activity might sustain a self-renewing, stress-resilient cytotoxic T cell pool capable of maintaining immune homeostasis.

## 4. Exercise-induced modulation of CD8⁺ T cell responses in cancer

Cancer represents the most comprehensively studied setting in which exercise modulates CD8⁺ T cell immunity, with mechanistic insights spanning metabolic reprogramming, cytokine signaling, and tumor-immune interactions. Accordingly, this section synthesizes the most detailed evidence available to date on how exercise shapes CD8⁺ T cell responses in malignant disease.

### 4.1 Exercise modulates CD8⁺ T cell immunity in precancerous conditions

**Figure 3:**
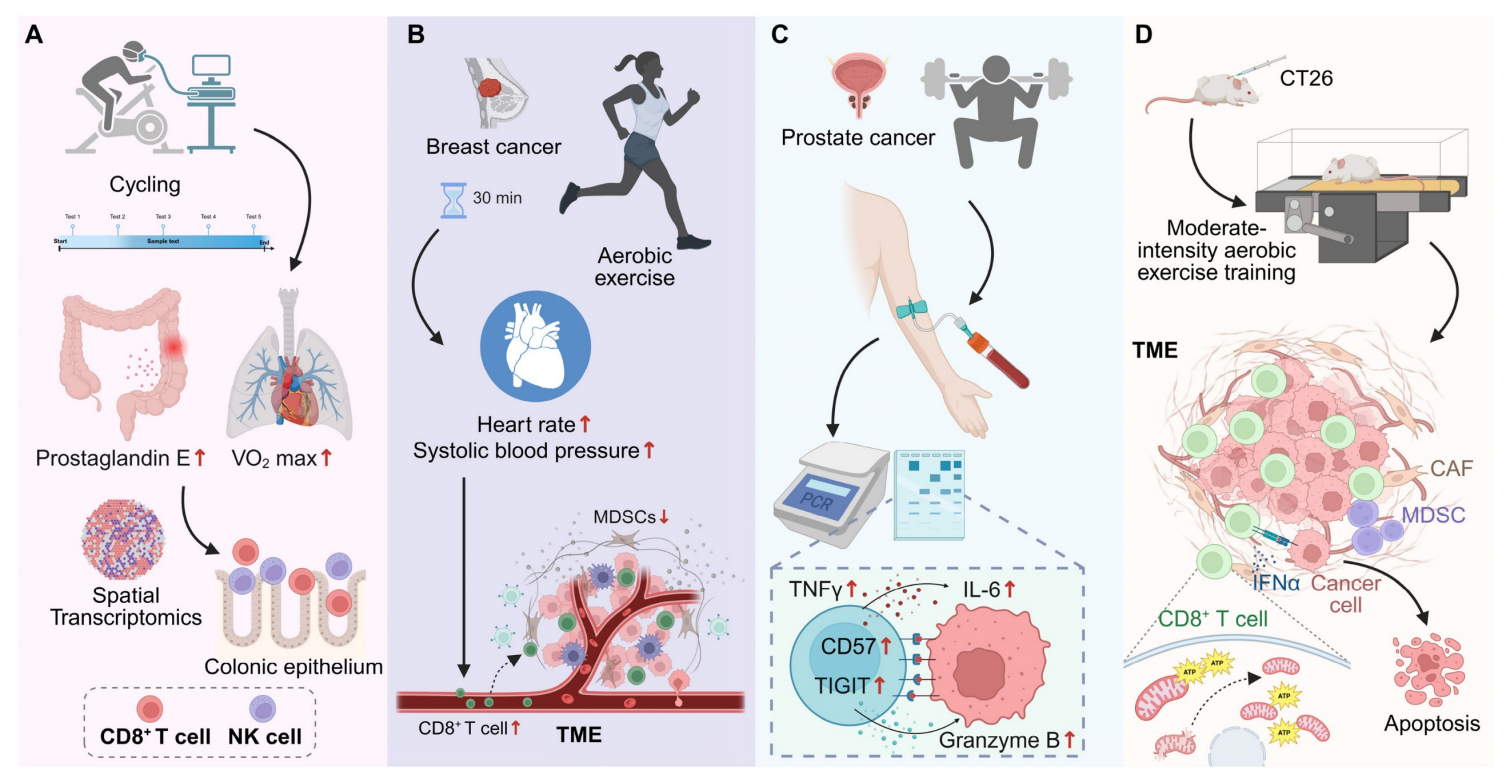
** Exercise modulates CD8⁺ T cell responses across precancerous and cancer contexts.** (A) In individuals with Lynch syndrome, long-term structured cycling enhances cardiorespiratory fitness, reduces colonic prostaglandin E levels, and promotes CD8⁺ T cell and NK cell infiltration into the colonic epithelium, as revealed by spatial transcriptomics. (B) In newly diagnosed breast cancer, a single session of moderate-intensity aerobic exercise transiently mobilizes circulating CD8⁺ T cells and NK cells, while reducing myeloid-derived suppressor cells, correlating with exercise-induced hemodynamic changes. (C) In prostate cancer, acute high-intensity exercise mobilizes CD8⁺ T cells with increased expression of CD57, TIGIT, and granzyme B, alongside transient inflammatory cytokine responses. (D) In mouse models, moderate-intensity aerobic training enhances CD8⁺ T cell infiltration and function within TME, preserves mitochondrial fitness, increases IFN-γ and ATP production, and promotes cancer cell apoptosis, thereby reducing tumor burden. Created in https://BioRender.com.

Physical activity is increasingly recognized as a physiological modulating cue that reshapes immune homeostasis and mitigates early tumorigenic risk, particularly in individuals with precancerous conditions 68-70. In Lynch syndrome—a hereditary disorder predisposing to colorectal cancer—aerobic exercise not only enhances cardiorespiratory fitness but also modulates CD8⁺ T cell immunity within the intestinal mucosa. A 12-month structured cycling regimen promotes sustained enrichment of cytotoxic CD8⁺ T cells in the colonic epithelium, accompanied by reduced prostaglandin E levels and diminished systemic and local inflammation. Transcriptomic and spatial analyses further reveal that regular exercise reconfigures the mucosal immune landscape toward an antitumor, CD8⁺ T cell-dominant phenotype, coupled with increased NK cell infiltration and activation signatures. These findings suggest that exercise acts not merely as an immunomodulator but as a physiological modulating signal that enhances cytotoxic readiness and tissue-resident immunity, thereby reducing malignant progression risk in individuals with Lynch syndrome 71 (**Figure 3A**).

### 4.2 Exercise-driven remodulating of CD8⁺ T cell trafficking and tumor infiltration

Recent findings highlight exercise as a physiological remodulating cue that shapes CD8⁺ T cell trafficking and infiltration within the TME. Rather than passively enhancing immune cell recruitment, exercise actively remodulates the migratory and homing behavior of cytotoxic T cells, facilitating their entry into tumor tissue and strengthening immune surveillance 72, 73. Epidemiological and translational studies show that habitual physical activity before cancer diagnosis selectively increases CD8⁺ T cell densities at the invasive front and tumor core in colorectal cancer, independent of age, sex, or stage—an effect not observed for total CD3⁺ T cells, underscoring the selective modulating of cytotoxic subsets 74. In breast cancer, even brief bouts of moderate-intensity exercise trigger a transient yet directed mobilization of CD8⁺ T cells and NK cells, accompanied by a reduction in immunosuppressive myeloid-derived suppressor cells. This pattern suggests exercise-induced recalibration of the peripheral immune pool toward cytotoxic dominance, modulated by tumor hormone receptor status 75. Similarly, a 10-minute acute exercise session increases circulating CD8⁺ T cells by approximately 34%, with mobilization tightly correlated to hemodynamic parameters such as systolic pressure and heart rate, implying a mechanistic link between vascular stress and T cell trafficking modulating 16 (**Figure 3B**). In lymphoma and early-stage prostate cancer, high-intensity exercise induces transient mobilization of CD8⁺ T cells expressing CD57, TIGIT, and Granzyme B, reflecting a shift toward effector modulating. In animal models, moderate-intensity training reduces tumor burden through enhanced CD8⁺ T cell frequency and improved CD4⁺/CD8⁺ balance, with negative correlations between cytotoxic T cell abundance and tumor mass 76. Collectively, these findings demonstrate that exercise exerts a dose- and intensity-dependent modulating effect on CD8⁺ T cell trafficking, promoting effective infiltration and immune orchestration within the TME.

### 4.3 Exercise-mediated restoration of CD8⁺ T cell functionality in cancer

Exercise functions as a remoduting signal that restores functional integrity to metabolically and epigenetically exhausted CD8⁺ T cells within tumors 77. In multiple myeloma, a six-month structured training program significantly reduces exhaustion marker expression (PD-1, TIGIT, TIM-3, LAG-3), reshaping the transcriptional and metabolic modulating of CD8⁺ T cells toward a rejuvenated cytotoxic state 78. Mechanistically, exercise-induced myokines and metabolites remodel central carbon metabolism in both mice and humans, augmenting mitochondrial respiration and effector cytokine production. Adoptive transfer of CD8⁺ T cells from exercised mice confers superior antitumor efficacy in untrained hosts, providing direct evidence that exercise-driven metabolic remodulating enhances cytotoxic potential and memory formation 79. Moreover, moderate aerobic exercise improves CD8⁺ tumor-infiltrating lymphocyte (TIL) function by preserving mitochondrial integrity and oxidative capacity. Enhanced IFN-γ release and ATP generation correspond with reduced mitochondrial loss and hypoxia within tumors, indicating that exercise modulates mitochondrial fitness and effector persistence in situ 80 (**Figure 3D**). Collectively, these findings illustrate that structured physical activity not only alleviates CD8⁺ T cell exhaustion but reinstates their functional modulating, transforming exercise into a non-pharmacological intervention capable of restoring antitumor immunity.

### 4.4 Exercise-induced remodulating of the TME and CD8⁺ T cell dynamics

**Figure 4:**
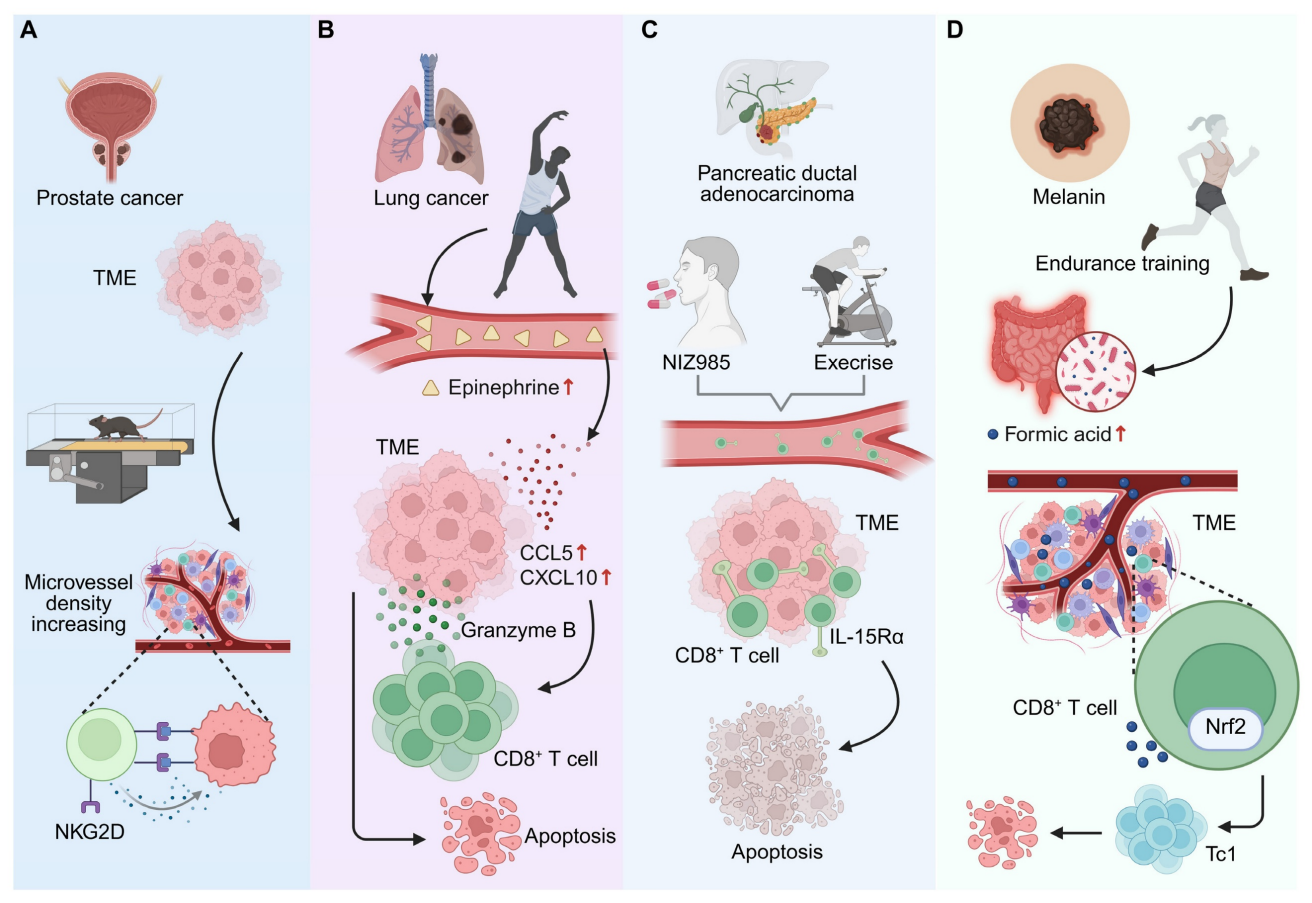
** Exercise-mediated modulation of tumor microenvironment and CD8⁺ T cell-related antitumor immunity.** (A) In prostate cancer, exercise increases microvessel density in TME, promoting NKG2D-related immune responses and potentially enhancing immune cell delivery to tumors. (B) In lung cancer, moderate-intensity exercise raises systemic epinephrine levels, which upregulate chemokines CCL5 and CXCL10 in the TME, recruiting CD8⁺ T cells and leading to tumor cell apoptosis. (C) In PDAC, exercise enhances CD8⁺ T cell mobilization and intratumoral accumulation via IL-15/IL-15Rα signaling. Pharmacologic activation with NIZ985 mimics these effects, enhancing chemotherapy and immunotherapy efficacy. (D) In melanoma, endurance training increases formic acid production in the gut microbiota. Formic acid activates Nrf2 in CD8⁺ T cells, promoting Tc1 differentiation and enhancing antitumor immune responses. Created in https://BioRender.com.

The TME critically governs immune responsiveness and tumor progression 81. Exercise functions as a systemic remodulating cue that remodels both vascular and immune niches within the TME, thereby reprogramming CD8⁺ T cell trafficking, persistence, and effector engagement. Aerobic exercise restructures the melanoma microenvironment through ERK5-dependent vascular normalization, enhancing perfusion and facilitating CD8⁺ T cell infiltration. In YUMMER tumors, this vascular-immune remodeling suppresses tumor growth, increases cytotoxic T cell accumulation, and induces phenotypic shifts in tumor-associated macrophages, including elevated MHC II expression 82. Similarly, voluntary running enhances antitumor immunity by increasing CD8⁺ T cell infiltration and NKG2D expression within tumors. Exercise generates a microvascular network characterized by higher density and improved regularity, which optimizes immune cell delivery and oxygenation. After 28 days of continuous exercise, tumors exhibit a marked rise in CD8⁺ T cell abundance, correlating with improved vascular function and reduced tumor mass 83 (**Figure 4A**). Notably, exercise-induced extracellular vesicles (EVs) extend this remodulating effect beyond systemic circulation. In triple-negative breast cancer, exercise-derived EVs transform the TME into a more immunologically active state by promoting CD8⁺ T cell infiltration and activation. Pre- and post-implantation administration of these EVs suppresses tumor growth in both EO771 and 4T1 models, particularly converting immune-excluded 4T1 tumors into T cell-responsive, inflamed phenotypes 84. These findings underscore that exercise-induced systemic cues—including EVs and vascular remodeling—modulate a permissive microenvironment for CD8⁺ T cell immunity, thereby transforming cold tumors into immunologically “hot” states and potentiating responsiveness to immunotherapy.

### 4.5 Exercise-induced neuroimmune remodulating enhances CD8⁺ T cell-mediated antitumor immunity

Accumulating evidence suggests that exercise engages neuroimmune cross-talk to remodulate the activation and trafficking of CD8⁺ T cells within tumors 85, 86. Moderate-intensity exercise elevates systemic epinephrine, which in turn upregulates chemokines CCL5 and CXCL10 in the TME, driving chemotactic recruitment and intratumoral accumulation of CD8⁺ T cells. This neuroendocrine-immune coupling results in significant tumor growth inhibition, an effect that can be recapitulated by exogenous epinephrine, confirming the β-adrenergic-dependent modulating of cytotoxic T cell migration and activation 87 (**Figure 4B**). Mechanistically, activation of β₂-adrenergic receptors during exercise mobilizes effector lymphocytes through cAMP-dependent signaling cascades that enhance TCR sensitivity and cytotoxic potential. Pharmacological modulation of this axis—specifically β₁-receptor blockade combined with PDE4 inhibition—further amplifies CD8⁺ T cell recruitment and effector activity 88. These observations position neuroimmune signaling as a dynamic modulating interface through which exercise fine-tunes the magnitude, localization, and efficacy of CD8⁺ T cell responses against tumors.

### 4.6 Exercise enhances tumor immunotherapy efficacy through CD8⁺ T cell modulating

Exercise amplifies the efficacy of cancer immunotherapy by remodulating CD8⁺ T cell metabolism, infiltration, and effector activation 14, 89. Through this integrative physiological conditioning, exercise enhances tumor immune surveillance and potentiates the action of immune checkpoint inhibitors 90. In pancreatic ductal adenocarcinoma (PDAC), exercise stimulates IL-15/IL-15Rα signaling, driving expansion and recruitment of CD8⁺ T cells into tumors and strengthening cytotoxic responses. Pharmacologic activation of this pathway using the IL-15 superagonist NIZ985 mimics these effects, sensitizing tumors to PD-1 blockade and augmenting chemotherapy efficacy 89 (**Figure 4C**). In colorectal cancer, acute exercise preceding immuno-chemotherapy accelerates early CD8⁺ T cell infiltration, producing a transient enhancement of immune efficacy that significantly reduces tumor volume during combination anti-PD-1 therapy 91. In breast cancer, exercise remodulates the tumor vasculature and immune milieu through the CXCL9/CXCL11-CXCR3 signaling axis, improving vessel perfusion, reducing hypoxia, and enabling sustained CD8⁺ T cell infiltration. This vascular-immune coordination renders previously resistant tumors responsive to PD-1 and CTLA-4 blockade 20. Beyond direct immune activation, exercise-induced metabolic remodulating via the gut microbiota contributes to the enhancement of CD8⁺ T cell-mediated immunity. In melanoma, exercise increases microbial production of formate, a metabolite that promotes Tc1 differentiation and cytotoxic function through Nrf2 activation. Elevated formate levels correlate with heightened antigen-specific CD8⁺ T cell activity and improved checkpoint inhibitor efficacy in vivo 92 (**Figure 4D**). Collectively, these findings demonstrate that exercise not only complements immunotherapy but modulates a metabolically and transcriptionally resilient CD8⁺ T cell response, thereby extending the therapeutic window for effective tumor control.

## 5. Exercise-induced modulation of CD8⁺ T cell responses in non-malignant diseases

While cancer provides the most extensively studied context for exercise-induced modulation of CD8⁺ T cell immunity, emerging evidence suggests that similar principles also extend to a range of non-malignant diseases. However, the depth and maturity of mechanistic insights vary considerably across these conditions. In this chapter, we summarize current knowledge on how exercise influences CD8⁺ T cell responses in infectious, neurological, and metabolic diseases, highlighting both shared immunological themes and areas where evidence remains preliminary. This broader perspective helps clarify the generalizability and limitations of exercise-induced CD8⁺ T cell modulation beyond cancer.

### 5.1 Exercise-induced CD8^+^ T cell mobilization and immune modulation in infection

By recalibrating the interplay between activation, memory formation, and exhaustion, exercise shapes CD8⁺ T cell functionality to sustain effective defense without excessive inflammation. This dynamic immunoregulation is reflected across distinct viral settings, where exercise intensity, duration, and context collectively influence the modulating of cytotoxic T cell responses. In influenza infection, an eight-week treadmill exercise program enhances resistance to influenza while dampening long-term CD8⁺ T cell memory formation in the lungs. Despite reduced granzyme B⁺ CD8⁺ T cell and antibody responses, exercised mice retain protection upon reinfection, indicating that moderate exercise recalibrates antiviral immunity by limiting inflammation yet preserving effective CD8⁺ T cell defense 93. Similarly, an eight-week endurance regimen augments antiviral responses by increasing CD8⁺ T cell infiltration into the lungs and reducing TNF-α production in influenza-specific cytotoxic cells. In obese hosts, exercise restores delayed immune activation, whereas in lean counterparts it promotes early viral clearance with restrained inflammation 94. These findings demonstrate that exercise intensity and metabolic context cooperatively modulate CD8⁺ T cell functionality to balance protection and tissue preservation during viral infection.

**Figure 5:**
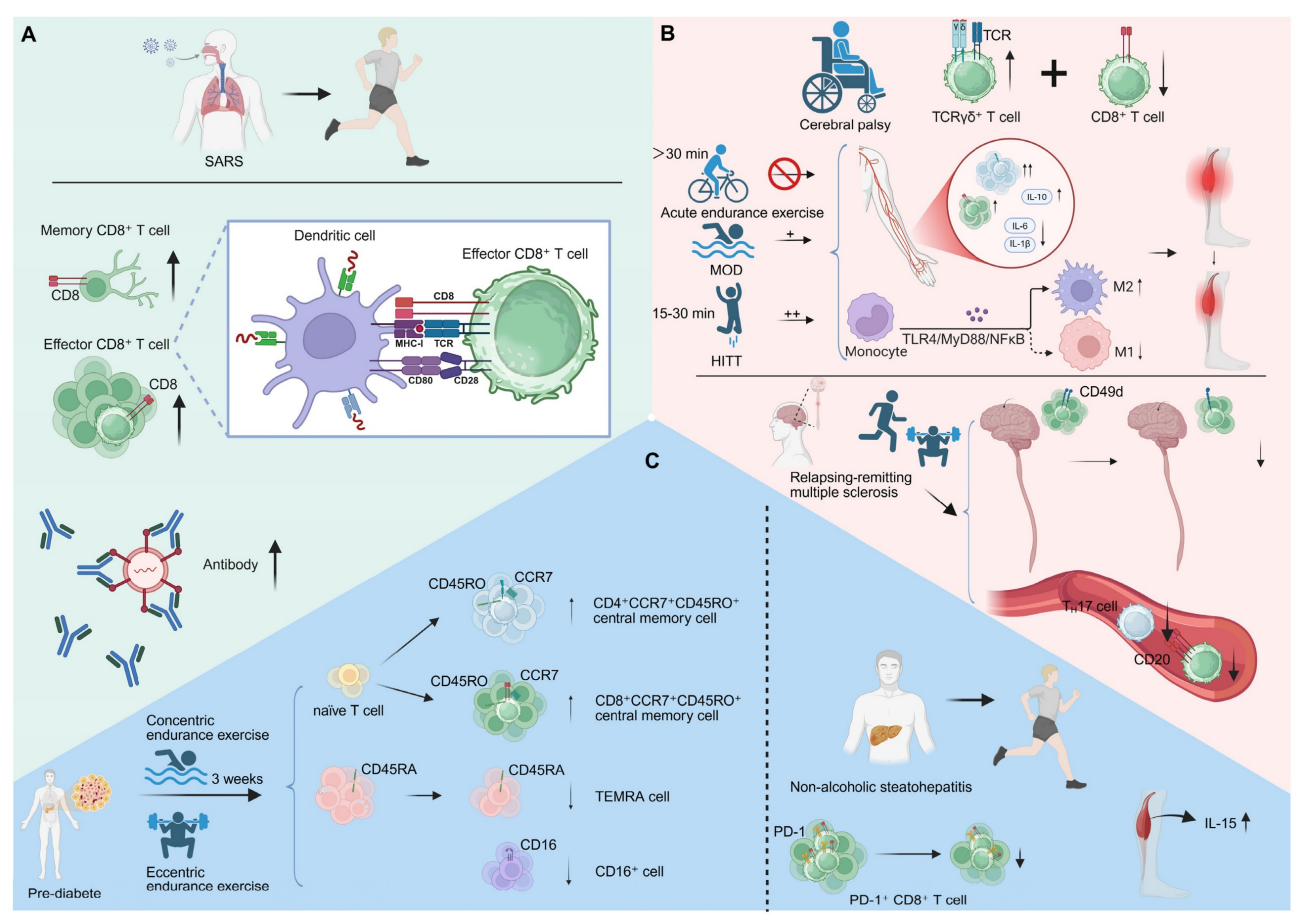
** Exercise-induced T cell mobilization and immune modulation in various conditions.** (A) Acute exercise mobilizes SARS-CoV-2-specific CD8⁺ T cells, increasing their circulation and enhancing immune surveillance. (B) In cerebral palsy, sufficient exercise intensity is needed for CD8⁺ T cell activation. HIIT enhances CD8⁺ T cell infiltration and function post-stroke and modulates macrophage activation. In relapsing-remitting multiple sclerosis, a 10-week multimodal exercise program reduces CD49d expression on CD8⁺ T cells and decreases proinflammatory Th17 cell levels. (C) In pre-diabetes, three weeks of concentric or eccentric endurance exercise reverses T cell senescence, increasing naïve and central memory T cells. In NASH, exercise reduces the accumulation of PD-1⁺ CD8⁺ T cells in the liver and elevates muscle - derived IL-15, mitigating chronic liver injury. Created in https://BioRender.com.

Emerging evidence in SARS-CoV-2 infection further supports this immune modulating paradigm. Acute exercise mobilizes a broad spectrum of SARS-CoV-2-specific CD8⁺ T cells, enhancing immune surveillance in individuals with natural immunity to the virus 95. Exercise induces a 2.5-fold increase in circulating functional CD8⁺ T cells, which recognize various viral peptides, including those from the spike, nucleocapsid, and membrane proteins. This response is further accompanied by a transient rise in SARS-CoV-2 neutralizing antibodies. Notably, exercise predominantly mobilizes MHC I-restricted CD8⁺ T cells, maintaining broad TCR-β diversity 96 (**Figure 5A**). Additionally, exercise rehabilitation restored exercise tolerance in post-hospitalised COVID-19 patients and enhanced immune homeostasis, as evidenced by increased naïve and memory CD8⁺ T cell subsets. Structured physical activity may thus remodulate CD8⁺ T cell dynamics toward a rejuvenated phenotype, suggesting that targeted exercise regimens can exert immunorestorative effects in post-COVID syndrome 97.

In persistent viral infections such as cytomegalovirus (CMV), regular combined exercise training rejuvenates CD8⁺ T cell immunity in CMV-seropositive older adults and enhances influenza vaccine responsiveness. These programs reduce CMV-specific immunoglobulins, restore naïve CD8⁺ T cell pools, and shift the effector-to-naïve balance, accompanied by elevated IL-10 and an anti-inflammatory milieu 98. In parallel, prolonged endurance training reverses infection-driven immunosenescence. A six-week strength-endurance regimen selectively reduces senescent T lymphocytes and expands naïve CD8⁺ populations in CMV-seropositive older women, reinforcing that sustained physical activity remodulates CD8⁺ T cell homeostasis and counteracts the age- and infection-related decline in immune flexibility 99. At the acute level, exercise intensity governs T cell redeployment, with vigorous exertion eliciting stronger mobilization and type I cytokine expression in CD8⁺ T cells. CMV infection further skews this response toward highly differentiated phenotypes, although its amplifying effect is independent of intensity 100. Together, these findings suggest that both acute and chronic exercise dynamically orchestrate CD8⁺ T cell trafficking, differentiation, and cytokine modulating, integrating metabolic stress and viral history to fine-tune immune activation.

In chronic active infections such as HIV, exercise exerts distinct immunomodulatory effects. A 12-week non-linear resistance training program enhanced immune competence in individuals living with HIV, elevating circulating CD4⁺ and CD8⁺ T cell counts while suppressing pro-inflammatory cytokines and increasing IL-10. This coordinated shift toward an anti-inflammatory and immunoregulatory profile indicates that structured resistance exercise can restore T cell homeostasis and counteract chronic immune activation in HIV infection 101. Notably, exhaustive exercise transiently mobilized mature and naïve T lymphocytes, yet HIV-infected individuals exhibited blunted CD4⁺ T cell recruitment and exaggerated CD8⁺ responses. This dysregulated T cell redistribution underscores impaired immune adaptability despite effective viral suppression, revealing that physical stress exposes persistent defects in T cell mobilization and homeostatic control in treated HIV infection 102.

### 5.2 CD8⁺ T cell-based immune modulation in neurological disorders induced by exercise

Exercise profoundly influences neuroimmune interactions, reshaping CD8⁺ T cell behavior across a spectrum of neurological conditions. Adults with cerebral palsy exhibit elevated baseline levels of TCRγδ⁺ T cells, indicative of chronic low-grade inflammation, and display a blunted CD8⁺ T cell mobilization following acute endurance exercise. The absence of post-exercise cytotoxic activation, despite comparable perceived exertion, likely reflects insufficient physiological intensity. These findings suggest that adequate exercise intensity is essential to modulate functional CD8⁺ T cell activation in individuals with cerebral palsy, underscoring the need for tailored immunometabolic interventions 103. Similarly, high-intensity interval training (HIIT) enhances CD8⁺ T cell infiltration and effector activity after stroke, outperforming moderate-intensity aerobic training. HIIT elevates the CD4⁺/CD8⁺ ratio and circulating IL-10 while suppressing proinflammatory cytokines (IL-1β, IL-6) and rebalancing macrophage polarization through the TLR4/MyD88/NFκB axis. These adaptations highlight that exercise intensity dynamically modulates T cell-macrophage crosstalk to restrain post-stroke inflammation and muscle atrophy 104. Moreover, a 10-week multimodal exercise program in individuals with relapsing-remitting multiple sclerosis remodulates CD8⁺ T cell immunity by reducing CD49d expression and limiting CNS homing. Exercise also lowers CD8⁺CD20⁺ and Th17 frequencies, collectively promoting a less encephalitogenic immune state. Such remodeling exemplifies exercise-driven modulating of CD8⁺ T cell trafficking and phenotype, ultimately dampening neuroinflammation and disease activity 105 (**Figure 5B**). A four-month moderate aerobic exercise program improved sleep and mood while recalibrating immune balance in patients with chronic insomnia. Exercise lowered circulating CD4⁺ and CD8⁺ T cell counts together with reduced cortisol levels, indicating a coordinated neuroendocrine-immune adjustment that restores physiological and immune equilibrium 106.

### 5.3 Metabolic disorders and exercise-induced CD8⁺ T cell modulation

Exercise has emerged as a potent regulator of immune homeostasis, capable of remodulating CD8⁺ T cell-mediated metabolic-immune interactions and attenuating the progression of metabolic disorders such as pre-diabetes and non-alcoholic steatohepatitis (NASH). 107, 108. In pre-diabetic individuals, three weeks of concentric or eccentric endurance exercise remodulates T cell immunity by reversing hallmarks of cellular senescence and reshaping the CD8⁺ T cell compartment. Exercise increases the proportion of naïve and central memory CD4⁺ and CD8⁺ T cells—particularly CD4⁺CCR7⁺CD45RO⁺ and CD8⁺CCR7⁺CD45RO⁺ subsets—while reducing terminally differentiated effector memory (TEMRA) and CD16⁺ cells. This coordinated remodeling suggests that exercise promotes the renewal and mobilization of naïve T cells while facilitating the clearance of senescent populations, thereby rejuvenating immune competence in pre-diabetes 109. Notably, a single bout of vigorous exercise selectively mobilizes differentiated CD8⁺ EMRA T cells; however, this response is markedly attenuated in individuals with type 1 diabetes. Reduced expression of adhesion and activation markers indicates defective trafficking and activation potential of these effector cells 110. Similarly, acute maximal exercise lowers circulating CD4⁺ T cells and the CD4⁺/CD8⁺ ratio while increasing NK-cell activity, yet fails to enhance CD8⁺ T cell recruitment in type 1 diabetes 111. These findings suggest that the capacity of exercise to modulate transient immune surveillance through CD8⁺ T cell redeployment is impaired in type 1 diabetes.

In a similar vein, exercise attenuates the progression of NASH by remodulating intrahepatic immune networks, particularly through reducing PD-1⁺ CD8⁺ T cell accumulation linked to liver inflammation and fibrosis. The decline in exhausted CD8⁺ T cells parallels improved hepatic architecture and suppressed proinflammatory cytokine production. Concurrently, elevated muscle-derived IL-15 orchestrates systemic immunometabolic communication. Collectively, these adaptations highlight exercise-driven remodulating of CD8⁺ T cell phenotypes as a pivotal mechanism mitigating chronic immune-mediated liver injury in NASH 112 (**Figure 5C**). Notably, an eight-week wheel-running exercise program restored bone integrity in ovariectomized mice by activating CD8⁺ T cells and elevating IFN-γ production. The resulting IFN-γ-dependent suppression of NF-κB and MAPK signaling inhibited osteoclastogenesis, linking exercise-induced CD8⁺ T cell activation to immune-mediated protection against bone loss 113 (**Table 2**).

## 6. Clinical evidence on exercise-mediated modulation of CD8⁺ T cell dynamics in aging, metabolic and cancer contexts

Clinical evidence increasingly indicates that exercise modulates immune function, particularly through the regulation of CD8⁺ T cell dynamics, metabolic fitness, and immune responsiveness. Structured training programs enhance immune surveillance and confer benefits across aging, metabolic and cancer contexts. A 12-week combined strength and endurance training remodulates CD8⁺ T cell immunity in older adults by constraining excessive differentiation and preserving a less-senescent phenotype. This intervention redirects kynurenine metabolism toward kynurenic-acid synthesis, mitigating inflammatory and neurotoxic stress. Collectively, these coordinated metabolic and phenotypic shifts indicate that structured exercise recalibrates immunometabolic circuits to sustain functional CD8⁺ T cell competence during aging 114. Both cardiorespiratory and resistance exercise interventions acutely remodel circulating CD8⁺ T cell pools (CD45RA⁺CD62L⁻ and CD57⁺CD45RA⁺CD62L⁻) in older adults. Resistance exercise expands cytotoxic populations in sedentary individuals, whereas endurance modalities preferentially enhance responsiveness in trained participants. These modality- and fitness-dependent effects illustrate how exercise parameters can be tuned to remodulate transient CD8⁺ T cell redeployment and optimize cytotoxic readiness 115.

In prediabetic individuals, a 12-week structured exercise intervention restores angiogenic CD31⁺ CD8⁺ T cell populations, linking improved metabolic capacity to vascular protection. This adaptive recalibration of CD8⁺ T cell subsets coincides with enhanced VO₂max and muscle strength, suggesting that exercise aligns immune and metabolic networks to counter cardiometabolic dysfunction 116. Consistently, in adults newly diagnosed with type 1 diabetes, a 12-week high-intensity interval training re-educates autoimmune CD8⁺ T cell activity by reducing the frequency and proliferation of islet-reactive clones. By restraining pathogenic CD8⁺ responses, exercise may preserve β-cell function and maintain glycaemic stability during the early autoimmune phase 117.

In localized prostate cancer, perioperative yoga training remodulates systemic immunity by increasing circulating CD8⁺ T cells and IFN-γ production while reducing regulatory T cells and myeloid-derived suppressor cells. This coordinated remodeling fosters an antitumor immune phenotype and exemplifies how exercise can modulate effector-suppressor balance 118. Likewise, in chronic lymphocytic leukemia (CLL), a 16-week physical activity intervention enhances fitness and reduces PD-1 expression on both CD4⁺ and CD8⁺ T cells, alleviating T cell exhaustion. The resulting higher CD4⁺:CD8⁺ ratio and improved cytotoxic competence suggest that sustained exercise reinvigorates modulated T cell functionality to support durable immune control 119.

Despite growing interest in this area, clinical evidence remains limited. Ongoing registered trials — including functional characterization of CD8⁺ T cells after supramaximal exercise (NCT06484101), exercise and COVID-19 viral T cell immunity (NCT05019456), and T cell responses to various levels of exercise stress (NCT06638684) — are expected to further clarify how exercise programs influence CD8⁺ T cell dynamics and immune resilience. However, as these trials have not yet reported results, current conclusions remain preliminary.

## 7. Challenges and future perspectives

Despite accumulating evidence that exercise can reshape CD8⁺ T cell-mediated immunity across diverse physiological and pathological settings, several unresolved challenges continue to limit its optimization and translation into clinical practice. These challenges span methodological uncertainties, biological complexities, and barriers to clinical implementation, each constraining the ability to design, monitor, and apply exercise regimens as precise immunomodulatory interventions.

Although exercise is increasingly recognized as a potent modulator of immune homeostasis, the precise “dose” required to maximize CD8⁺ T cell-mediated benefits without inducing adverse effects remains poorly defined. Immune adaptation to exercise follows a non-linear, context-dependent curve in which both insufficient and excessive intensity can blunt or even reverse immunological gains. Preclinical evidence illustrates this complexity. In breast cancer models, only moderate-intensity training, unlike low- or high-intensity regimens, enhanced antitumor immunity by increasing CD8⁺ T cell infiltration and effector function, including elevated proliferation and expression of CD69, IFN-γ, and granzyme B, in parallel with reduced tumor burden 120. While both low- and moderate-intensity exercise normalized tumor vasculature, only the latter augmented CD8⁺ T cell-mediated responses, indicating that vascular remodeling alone is insufficient to drive immune activation without appropriate intensity-dependent cues. Similar patterns are observed in human studies. In older women at high risk of breast cancer, moderate-intensity aerobic training increased circulating CD8⁺ effector memory T cells and reduced highly differentiated EMRA cells, suggesting attenuation of immunosenescence, with these changes correlating to improved cardiorespiratory fitness and increased β₂-adrenergic receptor expression on central memory CD8⁺ T cells. In contrast, high-intensity interval training decreased total CD4⁺ T cells and naïve subsets, potentially accelerating immune aging 121. The challenge lies not only in the biological non-linearity of these responses but also in the methodological heterogeneity of defining intensity, ranging from percentage of VO₂max to lactate threshold or perceived exertion, which impairs cross-study comparability and precludes precise meta-analysis. Moreover, physiological workload does not necessarily equate to immunological load, as individuals matched for relative intensity can exhibit divergent T cell mobilization kinetics and cytokine responses depending on baseline inflammatory tone, hormonal milieu, or disease state. Addressing this challenge requires harmonized frameworks that couple continuous physiological monitoring such as heart rate variability, oxygen uptake kinetics, and lactate clearance with longitudinal immune phenotyping, encompassing naïve-to-memory T cell ratios, activation markers including HLA-DR and CD69, and effector molecule production. Stratified multi-centre trials that integrate these metrics, combined with machine learning-based modeling, could generate individualized intensity-response curves, enabling both rigorous cross-study standardization and the development of dynamically adaptive, biomarker-guided exercise prescriptions tailored to specific immune profiles and disease contexts.

The immunological consequences of exercise are profoundly shaped by the cellular and metabolic architecture of the disease microenvironment, making the direction and magnitude of CD8⁺ T cell modulation highly unpredictable. Identical exercise regimens can yield diametrically opposed effects depending on immune composition, stromal organization, vascular integrity, and metabolic constraints. In EO771 breast tumors, exercise alone reduces both CD8⁺ T cell abundance and their proportion among total T cells, yet synergizes with anti-PD-1 therapy to enhance CD8⁺ T cell infiltration 122. In contrast, B16-F10 melanomas show minimal CD8⁺ T cell modulation with either intervention 123. These findings highlight that the impact of exercise on cytotoxic T cell responses is context-dependent and may enhance checkpoint blockade efficacy in immunotherapy-refractory tumors. Addressing this challenge requires a shift from one-size-fits-all prescriptions to microenvironment-informed exercise design. Pre-intervention profiling of the immune-metabolic state—quantifying inflammatory mediators such as IL-6 and TNF-α, immune checkpoint expression patterns, oxygenation status, and metabolite availability—could be used to classify patients into predicted responder or non-responder categories. Such biomarker-guided stratification would enable tailoring of exercise parameters, including intensity, duration, and modality, to the underlying immune context. Iterative monitoring during intervention cycles, leveraging circulating immune phenotyping, tumor-derived extracellular vesicle analysis, and non-invasive metabolic imaging, would allow dynamic adjustment of regimens as the microenvironment evolves. Integrating these data streams into predictive computational models could ultimately permit the generation of individualized, adaptive exercise prescriptions that account for microenvironmental heterogeneity and maximize beneficial T cell modulation across diverse disease states.

While exercise exerts multifaceted benefits on immune surveillance and T cell function, its physiological effects can also generate conditions that transiently or chronically compromise immune homeostasis. Sympathetic activation during acute exercise promotes adrenaline-mediated CD8⁺ T cell trafficking into peripheral tissues and tumor sites, yet this same adrenergic surge can trigger transient elevations in pro-inflammatory cytokines, potentially exacerbating inflammation in vulnerable individuals 88. Similarly, moderate increases in tissue perfusion enhance oxygen and nutrient delivery, facilitating immune cell migration and antigen presentation, but abrupt hemodynamic fluctuations in patients with advanced atherosclerosis or vascular aneurysms may precipitate cardiovascular events 124. At the redox level, physiological levels of reactive oxygen species (ROS) generated during exercise act as secondary messengers to promote T cell activation, mitochondrial biogenesis, and adaptive immune responses, whereas excessive ROS induces oxidative stress, DNA damage, and telomere attrition, accelerating immunosenescence 125. These dualistic effects highlight the need for risk-benefit calibration that considers both the intended immunological gains and systemic physiological vulnerabilities. Beyond biomarker monitoring, incorporating pre-intervention cardiovascular screening, individualized progression in training load, and concurrent use of redox-buffering nutritional strategies may reduce collateral risks while preserving immune benefits.

The translation of exercise-induced T cell modulation into clinical strategies is hindered by significant methodological and population biases. Much of the existing evidence derives from young, healthy individuals or animal models, with underrepresentation of older adults, patients with chronic diseases, and immunocompromised populations—the very groups most likely to benefit from immune support. This gap limits the external validity of current findings. Compounding this is the temporal complexity of T cell kinetics: mobilization, activation, and homing follow tightly regulated time courses, with peak effects often occurring within hours of exercise and rapidly resolving thereafter. Low-frequency sampling in clinical studies fails to capture these transient but functionally critical windows. Furthermore, the overwhelming reliance on peripheral blood measurements overlooks tissue-level dynamics, particularly within TMEs or sites of chronic inflammation, where the immune context may differ fundamentally from systemic circulation. Overcoming these barriers will require the development of high-resolution, minimally invasive monitoring platforms—such as wearable biosensors for cytokines, microfluidic devices for continuous immune cell profiling, and imaging modalities capable of capturing in situ T cell behavior. Longitudinal, disease-specific cohort studies incorporating such technologies will be essential to map the true spatiotemporal landscape of exercise-induced immune adaptation.

Despite promising immunological signals, the pathway from exercise-induced biomarker changes to clinically meaningful outcomes remains incompletely defined. The minimal effective “dose” of exercise necessary to sustain T cell rejuvenation is unknown, and patient adherence is frequently suboptimal, especially among those with physical limitations, fatigue, or treatment-related side effects. Without clear dose-response data, clinicians face difficulty in prescribing exercise regimens with predictable outcomes. More critically, improvements in surrogate immunological endpoints—such as increased naïve T cell frequencies or reduced regulatory T cell proportions—do not consistently correlate with hard clinical endpoints, including progression-free survival, relapse rates, or quality of life. This uncertainty limits exercise adoption as a formal adjunct to therapeutic protocols. Addressing these gaps will require randomized controlled trials that are sufficiently powered and of adequate duration to assess both immunological and clinical endpoints in parallel. Innovative trial designs could embed adaptive intervention arms, testing combinations of exercise with pharmacological or nutritional adjuncts to identify synergistic strategies that optimize patient adherence and maximize translation into durable clinical benefit.

## 8. Conclusion

The evidence synthesized here positions physical exercise as a systems-level regulator of CD8⁺ T cell biology, linking acute mobilization and trafficking to durable remodeling of subset composition, metabolic resilience and effector potential across both physiological and pathological contexts. By placing CD8⁺ T cells at the core of immune surveillance and tissue protection, this Review integrates mechanistic and translational perspectives to define when and how exercise can be leveraged to reinforce cytotoxic immunity. Mechanistically, convergent neuroendocrine, vascular and metabolic signals recalibrate chemokine gradients, activation thresholds and energetic supply, thereby shaping clonal expansion, memory formation and cytotoxic output. These adaptations encompass the rapid recruitment of activated and differentiated CD8⁺ subsets, preservation of mitochondrial competence, and attenuation of immunosenescence with sustained training, although their magnitude and direction remain highly contingent on host condition and the immune-metabolic architecture of target tissues.

Translating these insights into clinical benefit requires resolution of key uncertainties surrounding dose-response relationships, context-specific outcomes and physiological trade-offs. Exercise intensity and duration do not consistently predict immunological load, and disease-specific microenvironments can invert or obscure expected responses, while the dualistic nature of exercise physiology demands careful calibration to avoid exacerbating inflammation or oxidative stress in vulnerable populations. Overcoming these challenges will depend on integrating continuous physiological telemetry with longitudinal immune phenotyping, stratifying interventions according to baseline immune-metabolic state, and iteratively adapting regimens based on biomarker feedback. Coupling such precision frameworks with rigorous clinical validation could transform exercise from a supportive measure into a modulatory immunotherapy, capable of augmenting CD8⁺ T cell-mediated protection in prevention, treatment and health-span extension.

## Figures and Tables

**Table 1:** The impact of different exercise states on the physiological functions of T cells.

Functional impact	Intensity	Outcomes	Mechanisms	Ref.
Mobilization	Acute exercise	Lateral-differentiated T cell subsets mobilization	Lateral T cell subsets have more β2-adrenergic receptors	49
Metabolism	HIIT	Increasing CD8^+^ T cell cytotoxicity decreasing glycolytic capacity	Increasing G-protein-coupled receptor activity	50
Mobilization	Moderate-to-vigorous exercise	Increasing CD69^+^/CD8^+^ T cells rising mitochondrial oxidative capacity	Upregulating glucose transporters	51
Metabolism	/	Increasing CD8^+^ T cell response inhibiting T cell inflammatory response	Enhancing mitochondrial dependency Reducing glucose dependency	52
Metabolism	Chronic exercise	Increasing mitochondrial mass and elevating VO_2_	/	18
Metabolism	Acute endurance exercise	Rising inflammation index shifting to OXPHOS	/	53
Immunosenescence	/	Increasing proportion of naive T lymphocytes	IL-15 elevation to promote naive T cell survival	55
Immunosenescence	Moderate exercise	Increasing the proportion of CD4⁺ T cells	Enhancing telomerase activity or increasing expression of telomere-stabilizing proteins	56
Immunosenescence	Water-based training	Enhancing proliferation and activity of CD4⁺ and CD8⁺ T cells	Increasing antioxidant enzyme production and reducing oxidative stress	57
Immunosenescence	Low-dose exercise	Increasing CD4⁺/CD8⁺ T cell ratio reducing inflammation	Promoting thymic output of CD4⁺ T cells reducing the need for VEGF.	58
Mobilization	Plank exercise	Increasing CD4⁺ T cells and CD8⁺ T cells	Increasing rate of blood circulation	59
Immunosenescence	Moderate exercise	Increasing proportion of naive cytotoxic T cells	/	60

**Table 2:** The impact of various exercise modalities on immune cell alterations, underlying mechanisms, and clinical significance in patients with diverse diseases.

Exercise intensity	Exercise duration	Disease type	Immune cell changes	Mechanism	Implication	Ref.
Aerobic cycling	12 months	Lynch syndrome	NK cells↑ CD8^+^ T cells↑	/	Enhancing intestinal mucosal immunity	71
Physical activity 3 times per week	/	Colorectal cancer	CD8^+^ T cells in tumor front and center↑	/	Shifting immune profile towards anti-tumorigenic state	74
Moderate-intensity	30 minutes	Breast cancer	NK and CD8^+^ T cells↑ MDSCs↓	Hemodynamic regulation	Promoting anti-tumorigenic immune response	16
High-intensity	/	Prostate cancer	NK, NKT-like, and CD8^+^ T cell concentration↑	Increasing TNF-α and IL-6 leads to mobilization of immune cells	Improving NK cell cytotoxic activity against cancer cells	126
Moderate-intensity	12 weeks	Breast cancer	CD4⁺/CD8⁺, CD3^+^/CD4^+^↑	/	Reducing tumor burden	76
Moderate-intensity	Six months	Myeloma	T cell exhaustion markers↓	/	Reducing immune exhaustion	78
Acute exercise	/	Mammary cancer	CD8^+^ T cells↑	Metabolites produced in skeletal muscle	Enhancing effector profile of CD8+ T cells	79
Moderate-intensity	1 hour/day 5 days/week	Colon cancer	CD8⁺ TIL infiltration↑	Decreasing mitochondrial loss and increasing effector function	Elevating IFN-γ and ATP production	80
Aerobic exercise	2 weeks	Melanoma	CD8^+^ T cell infiltration↑	Modulating ERK5S496 phosphorylation	MHC II upregulation	82
Voluntary running	14 or 28 days	Prostate cancer	CD8^+^ T cells↑ NKG2D receptors↑	Improving blood perfusion	Regulation of tumor vasculature	83
Moderate-intensity	2 weeks	Breast cancer	CD8^+^ T cells↑	Extracellular vesicles ignite inflammation	Shifting TNBC tumors towards inflamed phenotype	122
Moderate-intensity	45 min/day 5 days/week	Lung cancer	CD8^+^ T cells↑ Epinephrine↑	Upregulating CCL5 and CXCL10	Inhibiting tumor growth	87
Moderate to high intensity	/	Lymphoma	Mobilization of lymphocytes and NK-cells	Increasing cAMP signaling	β2-adrenergic receptor activation	88
Moderate intensity	6 weeks	PDAC	CD8^+^ T cells infiltration↑ NK cell activity↑	Engagement of IL-15/IL-15Rα axis	Enhancing sensitivity to chemotherapy	89
Acute exercise	1 week	Colorectal cancer	CD8^+^ cytotoxic T cell infiltration↑	/	Improving effectiveness of immunotherapy	91
Moderate to high intensity	7-14 days	Breast cancer	CD8^+^ T cells infiltration↑ exhausted T cells↓	Activating CXCL9/CXCL11-CXCR3 signaling axis	Inducing vascular normalization	20
Moderate intensity	/	Melanoma	Tumor antigen-specific Tc1↑	Formating activates Nrf2 to promote Tc1 differentiation	Enhancing Tc1-mediated ICI efficacy	92
Acute exercise	20 min	SARS-CoV-2 infection	CD4^+^ T and CD8^+^ T cells↑	Catecholamine and β2-adrenergic receptor activation	Increasing SARS-CoV-2 immunosurveillance	96
Acute endurance exercise	45 min	Cerebral palsy	TCRγδ^+^ T cells↑ CD8^+^ T cells↑	Regulation cytokine production and T cell receptor expression	Enhancing immune responses to infections	103
High-intensity interval training	3 weeks	Muscle atrophy	M1 and M2 type macrophages↑	Downregulating TLR4/MyD88/NFκB signaling	Improving muscle repair and physical function	104
Multimodal exercise	10 weeks	Multiple Sclerosis	Frequency of Th17 cells↓	Reducing CD49d expression	Decreasing CNS-homing potential of CD8^+^ T cells	105
Endurance exercise	3 weeks	Pre-diabetic	CD4^+^/CD8^+^↑ CD4^+^/CD3^+^↑	Stimulating production and mobilization of naïve T cells	Reversing hallmarks of T cell senescence	109
High-intensity interval training	60 min/day 5 days/week	Nonalcoholic steatohepatitis	PD-1^+^CD8^+^ T cells↓	Inhibiting TLR4/MyD88/NFκB signaling pathway	Altering intrahepatic immune cell profile	112

## Abbreviations

MHC: Major histocompatibility complex
FOXP3: Forkhead box p3
CXCR3: C-X-C motif chemokine receptor 3
CCR5: C-C motif chemokine receptor 5
ICAM-1: Intercellular adhesion molecule 1
VCAM-1: Vascular cell adhesion molecule 1
EVs: extracellular vesicles
EM: Effector memory
PDAC: pancreatic ductal adenocarcinoma
VEGF: Vascular endothelial growth factor
TME: Tumor microenvironment
HIIT: High-intensity interval training
NASH: Non-alcoholic steatohepatitis
COPD: Chronic obstructive pulmonary disease
RA: Rheumatoid arthritis
CLL: Chronic lymphocytic leukemia
ROS: Reactive oxygen species
